# Differential Changes in Early Somatosensory Evoked Potentials between the Dominant and Non-Dominant Hand, Following a Novel Motor Tracing Task

**DOI:** 10.3390/brainsci10050290

**Published:** 2020-05-14

**Authors:** Mahboobeh Zabihhosseinian, Ryan Gilley, Danielle Andrew, Bernadette Murphy, Paul Yielder

**Affiliations:** Faculty of Health Sciences, University of Ontario Institute of Technology, 2000 Simcoe St. North, Oshawa, ON L1H 7K4, Canada; Mahboobeh.Zabihhosseinian@ontariotechu.ca (M.Z.); Ryan.Gilley@ontariotechu.ca (R.G.); Danielle.andrew@ontariotechu.ca (D.A.); Paul.yielder@ontariotechu.ca (P.Y.)

**Keywords:** somatosensory evoked potentials, motor acquisition, sensorimotor integration, dominant hand, non-dominant hand

## Abstract

During training in a novel dynamic environment, the non-dominant upper limb favors feedback control, whereas the dominant limb favors feedforward mechanisms. Early somatosensory evoked potentials (SEPs) offer a means to explore differences in cortical regions involved in sensorimotor integration (SMI). This study sought to compare differences in SMI between the right (Dom) and left (Non-Dom) hand in healthy right-handed participants. SEPs were recorded in response to median nerve stimulation, at baseline and post, a motor skill acquisition-tracing task. One group (*n* = 12) trained with their Dom hand and the other group (*n* = 12), with their Non-Dom hand. The Non-Dom hand was significantly more accurate at baseline (*p* < 0.0001) and both groups improved with time (*p* < 0.0001), for task accuracy, with no significant interaction effect between groups for both post-acquisition and retention. There were significant group interactions for the N24 (*p* < 0.001) and the N30 (*p* < 0.0001) SEP peaks. Post motor acquisition, the Dom hand had a 28.9% decrease in the N24 and a 23.8% increase in the N30, with opposite directional changes for the Non-Dom hand; 22.04% increase in N24 and 24% decrease in the N30. These SEP changes reveal differences in early SMI between Dom and Non-Dom hands in response to motor acquisition, providing objective, temporally sensitive measures of differences in neural mechanisms between the limbs.

## 1. Introduction

The upper limbs allow us to dynamically interact with our environment, which is dependent upon the surroundings in which we are operating in, and the efficiency with which the limbs adapt to change. This adaptation is facilitated by the ability of the brain to recall previous experiences and compare these to potential state changes. Many studies have explored differences in adaptation strategies between the limbs, and the neural mechanisms that govern them has been collectively referred to as “handedness” [1]. Previous studies have revealed that the limbs utilize different control mechanisms when adapting to novel environments [1,2,3,4,5,6]. 

The central nervous system (CNS) relies on feedforward or predictive and feedback or reflexive control mechanisms, in order to plan motor commands for movement. These control mechanisms are based on the coordination of both visually observed consequences of the motor command and its proprioceptive feedback [7]. Routine daily behavior requires a variety of motor skills that have been acquired gradually through practice and interactions with the environment. During hand or limb movement, predicted sensations are compared to what is actually occurring; if there is a match between the predicted and the actual consequences, the pattern is maintained for future movements. However, if there is a mismatch between the two, this requires a correction and calibration of the forward model that the cerebellum relies on, to coordinate skilled movement and develop an accurate body schema [8,9].

It is accepted that dominant (Dom) limb control relies on predictive control mechanisms and therefore preferentially utilizes a feedforward internal model to compensate for external forces. In contrast, the non-dominant (Non-Dom) limb is more reliant upon feedback mediated impedance control mechanisms that are better at maintaining consistent and more stable movements [1,2,4,5,10]. The Non-Dom limb is more accurate at performing certain motor tasks, likely due to these feedback mechanisms [1,2,11]. A study by Yadav in 2011 utilized a fast point-to-point single-joint elbow-extension movement task and demonstrated that there were early shifts for left arm movements, which rely on feedback mechanisms, and late shifts for right arm movements, which rely on feedforward mechanisms. This is indicative of the fact that with a new skill, we are more reliant on those mechanisms which are consistent and stable, however, the later shifts rely on predictive mechanisms, due to the more innate nature of the task once it is well learned [3].

Feedback or impedance control is normally seen as a less efficient way to complete a task because of the higher cost of energy to the system, however it is generally seen to be more accurate than predictive feedforward mechanisms applied by the Dom hemisphere, as feedforward strategies require accurate representations of body and task dynamics [3]. These performance differences are reflected in physiological variances. For instance, the Non-Dom hemisphere has been found to be more excitable than the Dom hemisphere for both left and right-handers, as measured by transcranial magnetic stimulation (TMS) input-output curves [12]. Another TMS study comparing both hands found that only the Dom hand showed a significant decrease in an input-output curve slope over two days of training, suggestive of a decrease in M1 excitability [13]. Although this is evidence of underlying differences in control mechanisms, the learning rates between the two hands were the same [13]. This is important, because if the learning rates are the same for both limbs, the differences in performance are related to the control of the movement rather than the learning. Performance retention differences over time between Dom and Non-Dom limbs also provide a means to understand whether differences in motor acquisition also impact subsequent retention, which is a way to ensure that motor learning has occurred. Many previous studies are difficult to compare, due to differences in study paradigms (e.g., use of exercise as a primer for learning) [14]. Asymmetric retention effects in which performance is enhanced with the Dom hand are typically observed [15].

A critical component of these control mechanisms is the accurate integration of sensory input to inform adaptive motor output. Motor skill learning tasks have demonstrated plastic changes within cortical and subcortical areas, as well as distinct interactions that occur between cortico-striatal and cortico-cerebellar systems [10,11]. An important area of the brain for sensorimotor integration (SMI) is the cerebellum, which integrates many forms of sensory feedback to mediate motor control and learning, with reciprocal influences between eye and body movements [16]. Cerebellar excitability modulates visuospatial and neurophysiological interactions that predict the tendency for efficient or compromised motor learning [17]. The cerebellum can modulate sensory inputs that create and modify motor responses based on expected sensory outputs [18], thereby directly influencing neural pathways between the cerebellum and the sensorimotor cortex that can be measured using short latency somatosensory evoked potentials (SEPs) [19].

Somatosensory evoked potentials (SEPs) are neurophysiological signals that have been used to investigate the integration of sensory and motor stimuli, as well as the effects of motor learning within the cortex [19,20,21,22,23,24,25]. Changes in various SEP peaks following a pre-, post- measurement protocol reflect changes in the excitability of different neural generators as a result of the intervention [20,23]. SEP changes provide a measure of the variations in synapse number or strength in widespread neural networks as a result of the learning process [26]. Studies have applied repetitive voluntary movements with SEP paradigms and demonstrated alterations in the processing of somatosensory information as evidenced by differences in SEP peak amplitudes following motor task performance [20,23,27,28,29,30]. Two SEP peaks of particular interest for this current study are the N30, which reflects changes in sensorimotor integration (SMI) pathways, and the N24, which reflects activation of neurons in the pathway between the cerebellum’s primary sensory area (SI), and is thus likely to reflect changes in cerebellar output [19,21,22,23,24,31,32,33]. The N24 SEP peak changes reflect the role of the cerebellum in sensorimotor prediction [34] and SMI [35]. 

Differential changes in SEP peak amplitudes would provide insight into some of the areas and processes that differ between the limbs that lead to different control strategies that are employed by the Dom and Non-Dom limb. This study therefore sought to explore the possible differences in early sensorimotor processing between the right (Dom) and left (Non-Dom) hand in healthy right-handed participants. It was hypothesized that the Non-Dom hand would have superior baseline motor performance of the novel task due to its stronger initial reliance on feedback processing, and that though the control strategies differ, the learning rates would be similar between the Dom and Non-Dom hands in post-acquisition and at retention. It was also hypothesized that the novel motor acquisition-tracing task would lead to differential changes in SEP peaks, reflecting early somatosensory processing and SMI between the hemispheres. 

## 2. Methods

### 2.1. Participants

Two groups of 12 healthy young right-hand dominant individuals with no known neurological conditions participated; each group was comprised of 6 males and 6 females, (mean age (standard deviation), and participants were randomly assigned to use either the right (Dom) hand group, or the left (Non-Dom) group. Group 1 (Dom), 19.87 years (0.99) and Group 2 (Non-Dom), 20.22 years (1.9). Group size was calculated using G-power statistical software, which indicated that for a repeated measures design with 2 groups, large effect size (f = 0.4), power (1 − β) = 0.95, and alpha of 0.05, that 12 participants per group would be needed [36]. Groups only completed the task with one limb, to avoid possible inter-limb transfer of information. All participants filled out a safety checklist and Edinburgh Handedness Inventory (EHI) self-report questionnaire [37]. Handedness was confirmed due to hemispheric differences in cortical excitability between Dom and non-Dom limbs in response to motor learning [13]. Written informed consent was obtained and the study was approved by the Ontario Tech University Research Ethics Board. This study was carried out according to the ethical standards set out by the Declaration of Helsinki for the use of humans in experimental studies.

### 2.2. Experimental Protocol

This study was a between-group experimental design, exploring differences in early SEP peaks following a motor acquisition task performed with either the right (Dom) or left (Non-Dom) hand in separate groups of healthy right-handed participants. All participants were required to attend two sessions, 24 h apart. While optimal retention has been observed 5–6 days after an initial training session, it has also been observed within a minimum of 6–8 h after training [38]. After applying all instrumentation, the first session started by obtaining the pre-SEP (baseline) measurements through the stimulation of the median nerve, as outlined in stimulation parameters. Participants then completed the motor acquisition task consisting of pre-acquisition (four tracing trials), acquisition phase (twelve tracing trials), and post-acquisition (four tracing trials). Then, post-SEP measurements were obtained. The second session measured retention using the same pre- and post- motor acquisition task performed in session one. During the second session, SEPs were not collected, as the purpose of this session was to determine whether motor learning had been retained. A schematic of the protocol design can be seen in Figure 2. 

### 2.3. Stimulation Parameters

SEP recording electrodes were placed according to the International Federation of Clinical Neurophysiologists (IFCN) guidelines [39]. Stimulation electrodes were placed over the median nerve at the wrist on either the Dom or the Non-Dom hand, between the tendons of flexor pollicis longus and palmaris longus. Recording surface EMG electrodes (Ag-AgCl, Meditate, conductive adhesive hydrogel) were placed: (1) on the ipsilateral brachial plexus (Erb’s point) for upper extremity SEPs, with the electrode placed posterior to the clavicle and as medial to the sternocleidomastoid as possible, without placing directly over top of the muscle; (2) Over C5 spinous process (Cv5), which was landmarked by starting from C7 and locating the ascending spinous processes; (3) anterior neck or trachea (supraglottic region on the midline). The cephalic site recoding EEG electrodes (10 mm disc, 2-mm hole gold cup EEG electrodes, Grass Technologies; Astro-Med; Subsidiary, Rockland, MA, USA; impedance < 5 kΩ), were measured from the vertex of each participant and were placed on parietal site (CC’) (20% of the subject’s tragus to tragus measurement and 2 cm posterior to contralateral to vertex or Cz), and frontal site (Fc’) (6 cm anterior and 2 cm contralateral to Cz) [32].

To minimize the electrical artifact, the ground electrode was placed in the mouth (between the stimulation site and the recording electrodes). The C5 spinous process was referenced to the trachea, while all other electrodes were referenced to the ipsilateral earlobe. Each of the sites was properly cleaned prior to electrode placement with shaving, abrasive pads, and alcohol swabs.

The SEP stimulation protocol consisted of the presentation of 200 µs electrical square pulses, delivered at the wrist over the median nerve. The stimuli were delivered at a constant intensity at two frequencies of 2.47 Hz and 4.98 Hz by Ag/AgCl EMG conductive adhesive surface electrodes (MEDITRACE^TM^ 130, Ludlow Technical Products Canada) (impedance < 5 kΩ). These different frequencies were used to accurately differentiate between the N30 and the N24 SEP peaks, as they often overlap each other, and at faster rates, the N30 diminishes and the N24 becomes more prominent, and easier to measure [30]. Stimulation was delivered at motor threshold for each participant, which causes a noticeable and constant twitch in the abductor pollicis brevis (APB) muscle of the thumb. SEP peak amplitudes were measured from the averaged 1500 sweeps of the waveforms.

The SEP signals were amplified (gain of 10,000), filtered (0.2–1000 Hz) using a configuration written in Signal software (Version 4.08, Cambridge Electronic Design, Cambridge, UK). The amplitudes of the SEP peaks were measured from the peak of interest to the earlier or later peak of opposite deflection [39]. The amplitudes and latencies of the following short-latency SEP components were identified and analyzed: the peripheral N9, the spinal N11 and N13, and the far-field N18 (P14–N18 complex), the parietal N20 (P14–N20 complex), and P25 (N20–P25 complex), the frontal N24 (P22–N24 complex), and the frontal N30 (P22–N30 complex). 

### 2.4. Motor Acquisition Tracing Task Parameters

The motor acquisition-tracing task was developed using a custom leap motion software tool (Leap Motion, San Francisco, CA, USA). Each trace was a sinusoidal waveform made up of a series of color-coding dots, with 500 dots equaling one trace, which moved vertically down a monitor, while the participant attempted to trace each dot as it passed the horizontal axis. The task consisted of four different sinusoidal waveforms that were presented in random order, which were designed to change in complexity through the variation and randomization of both the frequency and amplitude at the starting, middle and ending of the each sinusoid, to allow for continuous improvement over time. Time was varied for each trial (37, 41, 49, and 83 s) and the speed was the same for all trials (70 pixels per second). Participants were trained to perform an abduction/adduction of the thumb, involving a sweep from left to right, utilizing the abductor pollicis brevis (APB) muscle. 

**Figure 1:**
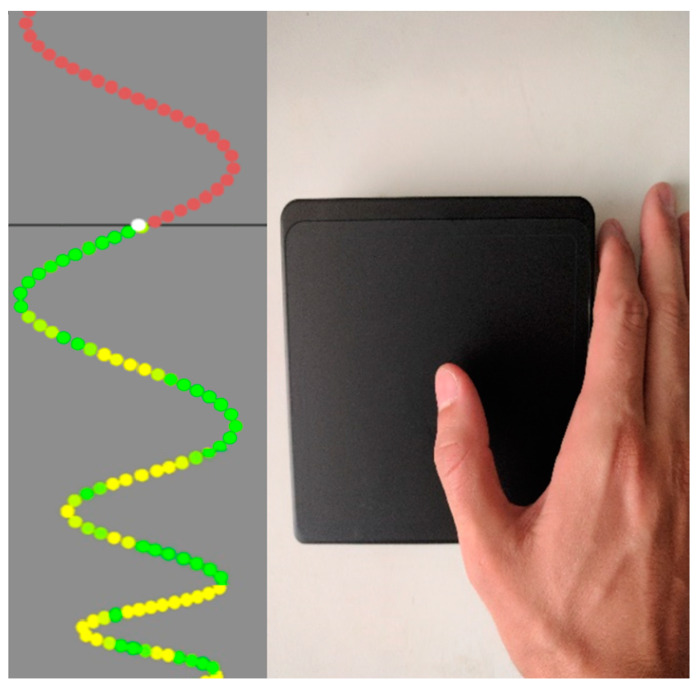
Motor acquisition tracing task (Custom Leap Motion Software Tool). (**Left**): Continuous sinusoidal waves move vertically down a monitor and participants attempted to copy each dot as it passed the black horizontal axis. Color-coded dots show trace accuracy, with the trace changing to green to represent good accuracy and yellow to represent less accurate performance. (**Right**): participant attempted to copy each dot as it passed the horizontal axis, using their thumb on an external touch pad.

Each sinusoidal waveform is close together at the beginning and get wider at the end of each trial. The participant was given color-coded feedback, with the trace changing to green to represent good accuracy, and yellow to representing less accurate performance (Figure 1). All participants could perform the simplest trace easily and the most difficult was challenging to all participants, with the other two traces of medium difficulty, as described in previous studies [13,21,23,40]. This allowed room for continuous improvement for all participants.

**Figure 2:**
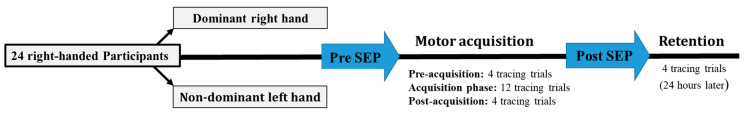
Experimental protocol.

For the pre-acquisition, post-acquisition, and retention trials, each of the four versions was performed once (approximately 3-min time duration), and for the acquisition phase, each version was performed three times (in random order), for a total of 12 traces (taking approximately 10-min) (Figure 2). Participants were instructed to replicate each trace as accurately as possible, using their thumb on an external wireless touch pad. The traces can only be followed in the left-right direction, eliminating up and down error. The participants were instructed to use little to no wrist or elbow action to replicate the trace, utilizing only the APB muscle. To ensure stability of the arm, to isolate the thumb movement during task performance, and to prevent shoulder fatigue, the participant’s forearms were rested atop adjustable armrests. Motor error was determined by calculating the average distance of the attempted trace from the original template trace and motor tracing improvement refers to a decline in the percentage of error throughout the task.

## 3. Data Analysis

Motor performance on the tracing task both pre- and post-motor acquisition and retention, and the amplitude of the early SEP peaks pre and post motor acquisition, were analyzed separately. The tracing task data (percentage error for each trial) was exported to Excel™. The percentage error represents distance from the dot on the trace, where 100% error equals one dot width (6.62 mm) in distance away from the template trace. For each participant, percentage error was averaged per trial for each trial in all test conditions in the pre-acquisition, post-acquisition and retention tests. SEP peak-to-peak amplitudes were measured pre- and post-motor acquisition task and latency was also checked, to determine if there were any changes in processing time or speed following the motor acquisition task. The peak-to-peak amplitudes were normalized relative to the baseline trial to allow comparison between groups. Once normalized, group averages were calculated to measure the proportional changes in pre- and post-motor acquisition SEP peak amplitudes. 

## 4. Statistical Analysis

### 4.1. Neurophysiological Data

All data were tested for homogeneity of variance and skewness to ensure parametric statistics could be run. SEP peak amplitudes were compared using a 2 × 2 mixed-design repeated measures analysis of variance (ANOVA) with time (pre- vs. post- motor acquisition) as the repeated measure and group (Dom vs. Non-Dom), as the between subjects factor for each SEP peak amplitudes. Post-hoc ANOVA (Bonferroni correction) were used to compare the pre- and post-intervention SEP differences within each group. It was important that trials included in this study had a peripheral N9 amplitude change of no greater than ±10%. Any changes greater than 10% would indicate changes in the incoming peripheral afferent volley, perhaps due to changes in posture, and a stable and consistent afferent volley is essential in order to be able to attribute changes in centrally generated SEP peak amplitudes to acquisition-induced plasticity. 

### 4.2. Behavioral Data

Independent samples t-tests were used to determine if there were any baseline differences between the Dom and the Non-Dom group. The accuracy of tracing task performance as defined by average percentage of error for time points of pre-acquisition, post-acquisition and retention were compared between the Dom and the Non-Dom group. The accuracy data were normalized to each individual’s baseline value, in order to enable comparison of the relative improvements in performance between groups, and a 2 × 3 mixed-design repeated measures ANOVA was run with time (pre-acquisition, post-acquisition and retention), as the repeated measure and group (Dom vs. Non-Dom) as the between factors. 

Statistical significance was set at *p* ≤ 0.05 for all analysis (SPSS v.25, IBM Corporation, Armonk, NY, USA). All numeric values are expressed as mean ± standard deviation (SD).

## 5. Results

All of the recruited subjects (*N* = 24) completed the study and were included in the SEP peak analysis. According to the inclusion criteria for SEP data analysis, there were no significant differences (*p* = 0.63) in the N9 SEP peak, and it differed less than ±10% between pre- and post-intervention trials in order for that participant’s data to be included. All participants were right hand dominant with the EHI value of 86.36 (14.12), females 89.22 (1.2) and males 83.33 (18).

### 5.1. Neurophysiological Data

**Figure 3:**
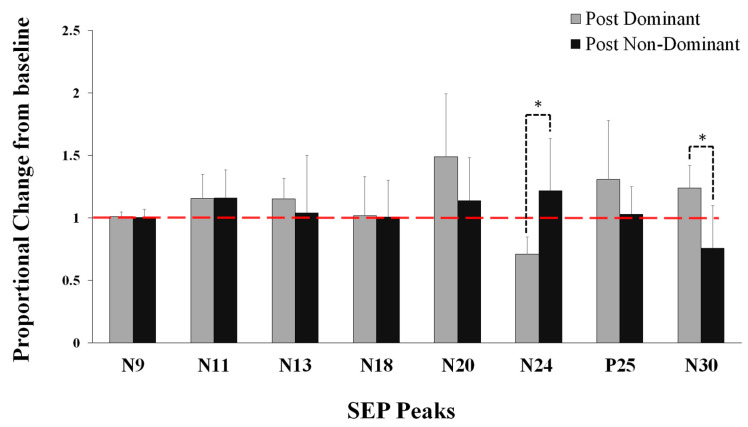
Averaged normalized somatosensory evoked potentials (SEP) peak amplitudes relative to baseline (dotted line), showing Dom vs. Non-Dom groups after motor acquisition tracing task. Note: the significant interactive effects between groups were seen for N24 and N30 SEP peaks (* *p* < 0.001). Error bars represent SD.

**Figure 4:**
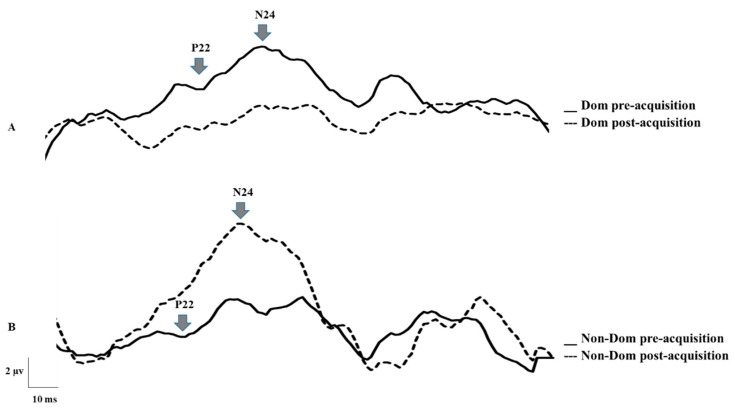
Raw data from an average 1500 sweeps of the waveforms of two representational participants. Pre- and post-motor acquisition N24 SEP peak amplitudes for: (**A**) Dom hand and (**B**) Non-Dom hand.

N24: Following motor acquisition, there was a significant interaction effect of TIME by GROUP (Dom vs. Non-Dom) (F1, 22 = 16.348, *p* = 0.001), partial eta squared (η^2^) = 0.426, with no effect of TIME (*p* = 0.591). The N24 decreased by 28.9% (13.73) for the Dom group (F1, 22 = 53.145, *p* = 0.0001) and increased none significantly by 22.04% (41.43) for the Non-Dom group (*p* = 0.079) (Figure 3 and Figure 4). 

N30: Following motor acquisition, there was a significant interaction effect of TIME by GROUP (F1, 22 = 18.687, *p* = 0.0001, η^2^ = 0.459), with no effect of TIME (*p* = 0.983). The N30 increased by 23.8% (18.1) for Dom group (F1, 23 = 20.743, *p* = 0.0001) and decreased by 24% (33.8) for Non-Dom group (F1, 23 = 6.072, *p* = 0.022) (Figure 3). 

There were no significant differences between groups for N11 (*p* = 0.955), N13 (*p* = 0.439), and N18 (*p* = 0.960), and P25 (*p* = 0.079). The N20 peak approached a significant effect of TIME by GROUP (F1, 22 = 3.931, *p* = 0.060, η^2^ = 0.152), with a significant effect of Time (F1, 22 = 12.75, *p* = 0.002, η^2^ = 0.367) (Figure 3).

### 5.2. Behavioral Data

**Figure 5:**
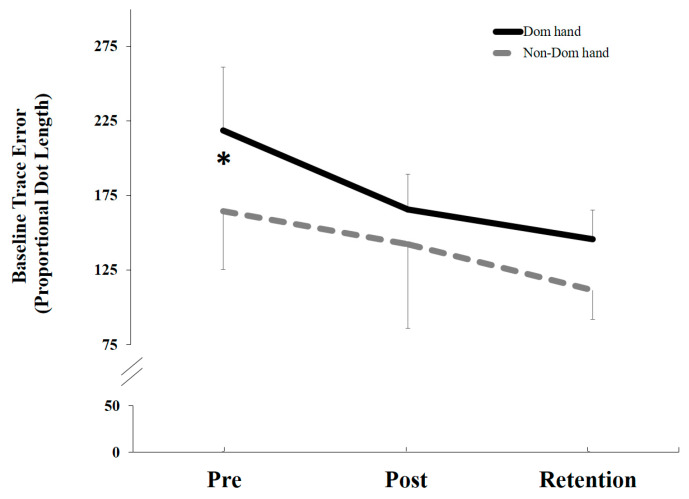
Average percentage error differences for pre-acquisition (baseline), post-acquisition and retention for both groups (Dom vs. Non-Dom). The Non-Dom group was significantly more accurate at baseline, relative to the Dom group (* *p* < 0.0001). Error bars represent SD.

The Non-Dom group was significantly more accurate at baseline (t(11) = 17.84, *p* = 0.0001), 164.63 (33), as compared to the Dom group 218.79 (44) (Figure 5). Since the two groups were different in baseline accuracy, to be able to directly compare changes in performance between the groups, the post-acquisition and retention accuracy data were normalized to the pre-acquisition data (Baseline). 

**Figure 6:**
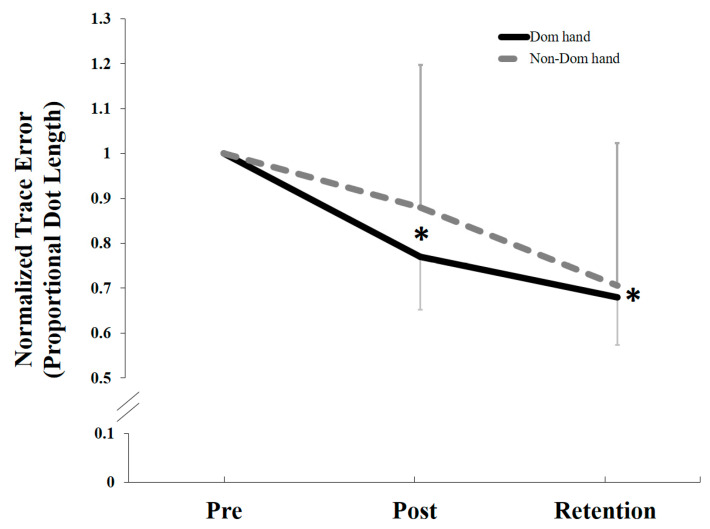
Normalized mean proportional trace error changes by groups (Dom vs. Non-Dom). Both groups improved in accuracy following motor acquisition and retention. There was a significant overall effect of TIME (* *p* < 0.0001), but no significant interaction effect TIME by GROUP. Error bars represent SD. * represents the significant differences from baseline over time.

Following the motor acquisition tracing task, there was a significant overall effect of TIME (F1, 22 = 25.82, *p* < 0.0001) for the normalized data, but no significant interaction effect of TIME by GROUP (Dom vs. Non-Dom) for both post-acquisition *p* = 0.276 and retention *p* = 0.664. Following both motor acquisition and retention, the Dom group improved by (23% post-acquisition and 32% at retention), as compared to the Non-Dom group (12% and 29%), respectively (Figure 6). 

### 5.3. Correlations between the Motor Skill Acquisition and SEP Peak (N24 and N30) Data

To explore the relationship between both neurophysiological and behavioral measures and the potential they have to be used together in understanding sensorimotor integration, a Pearson’s correlation between neural activity (post SEP) and motor task performance (accuracy post-acquisition phase) was performed. Statistical significance was set at *p* = 0.05.

**Figure 7:**
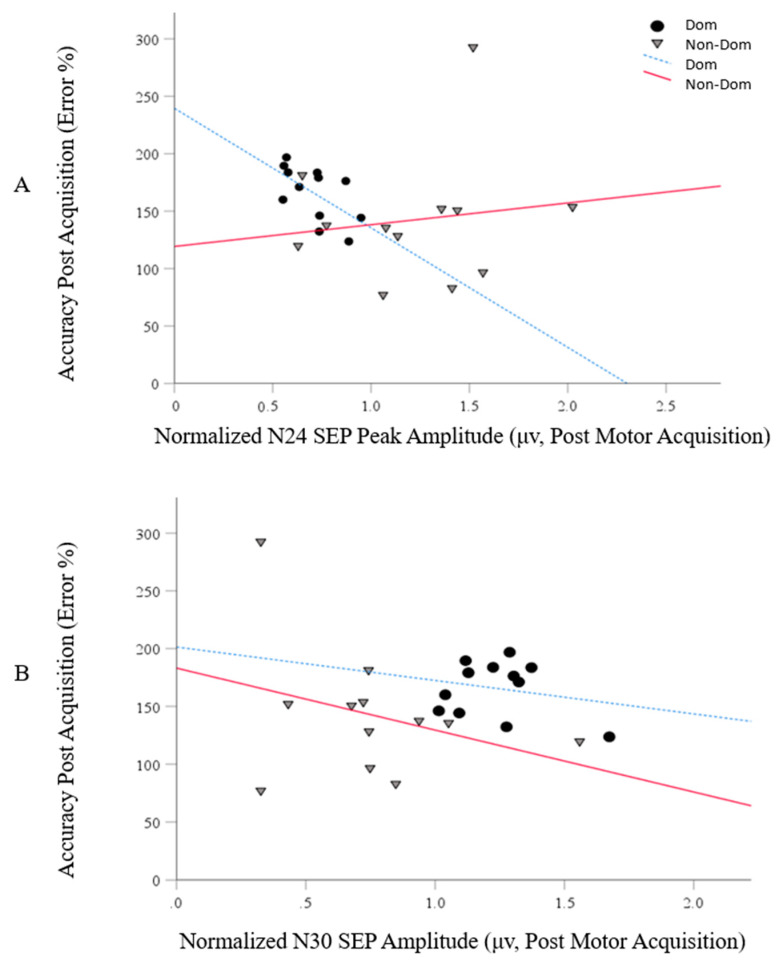
Graphical representations of the correlations for SEP peaks. Panel (**A**) is for the N24 and panel (**B**) is for the N30.

Significant negative correlations were reported for the N24 peak and behavioral performance (*r* = −0.599, *p* = 0.02), with no correlation observed for the N30 peak (*r* = −0.22, *p* = 0.26) for those within the DOM group. In addition, no significant correlations were regarded for the N24 (*r* = 0.139, *p* = 0.333) and the N30 peak (*r* = −0.04, *p* = 0.450) within the NON-DOM group. These correlations are represented in Figure 7. 

## 6. Discussion

This study investigated the effect of motor acquisition on sensorimotor processing by measuring changes in early SEP peak amplitudes between Dom and non-Dom hands, following performance of a motor acquisition-tracing task. Differential changes were evoked in early SEP peaks related to SMI in the Dom vs. Non-Dom group following motor acquisition. The amplitude of the N24 SEP peak significantly decreased for the Dom group and increased for the non-Dom group. In addition, the N30 SEP peak amplitude significantly increased for the Dom group and decreased for the Non-Dom group. There were significant improvements in accuracy for both groups both post-acquisition and at retention, suggesting that motor learning occurred. Additionally, the Non-Dom hand was more accurate at baseline compared to the Dom group. Significant negative correlations were reported for the N24 peak and behavioral performance within the Dom group. 

The directional differences in peak amplitude changes between the limbs may be the result of different neural pathways that reflect a selective way to produce movement. Evidence of sensorimotor loops within the posterior parietal cortex (PPC), at the area of the intraparietal sulcus (IPS), and the cerebellum suggest the use of both feedforward and feedback control mechanisms associated within these structures [41]. These loops rely on a feedforward model that integrates the sensory inflow and motor outflow to understand and examine the consequence of the motor commands utilized by the limbs, and allows the command to unfold with the guidance of internal and external feedback [41]. 

The N30 peak has been demonstrated to represent activation in the premotor, motor and prefrontal areas of the cortex; this is supported by source localization studies indicating that these areas are involved in the generation of this peak [42]. The combination of these areas suggest a role in movement planning and SMI [21,42,43]. The decrease in the N30 peak for the Non-Dom hand following motor acquisition suggests that the areas associated with SMI and motor planning were less active in the early processing phase when compared to the Dom hand. It also represents an increase in inhibition within the pathway as learning occurred. This suggests that in order to produce the tracing task movements, information was inhibited online, indicative of feedback-based impedance control. However, the dominant limb showed an increase in peak amplitude following the tracing task. This may be the result of a decrease in inhibition, or an increase in excitability in SMI and motor planning areas. Both of these findings are consistent with the literature, which has found differences in feedforward control strategies utilized in the limbs [1,2,10].

The N24 peak is thought to reflect activation changes in the neural pathways between the cerebellum and S1 [31,33]. Many studies have shown that the cerebellum plays an active role in motor learning [12,19,21,22,23,44,45] and the changes in cerebellar-S1 pathways support these findings in that following the motor acquisition task, both groups showed changes in the activity of this pathway. Following the tracing task, the Non-Dom group had an increased N24 peak amplitude, suggestive of an increase in cerebellar inhibition, as the cerebellum uses inhibition to learn the motor program demonstrated in the task. The decrease in N24 amplitude seen in the Dom group may reflect a decrease in cerebellar inhibition. This would be expected, as the Dom limb relies more on feedforward processes, which would have to be “disinhibited” in order to learn a new skill. Additionally, the significant negative correlation for the N24 peak and behavioral performance within the DOM group reiterates the role that the cerebellum plays in SMI and decreased dependence on cerebellar activity when a novel task is well learned and there is not as much need to adapt or correct for performance errors [46]. This finding is supported by previous TMS work, showing a decrease in cerebellar inhibition following novel motor acquisition [44].

Previous research has shown that following a reaching task, the non-dominant limb seemed to follow a movement pattern specialized for stability and utilized feedback information more efficiently, while the dominant limb followed a movement pattern generated from an internal model and utilized feedforward information more efficiently [1,2,11,47]. A previous TMS study demonstrated that the non-dominant hemisphere of both left- and right-handed individuals was found to be more excitable at rest, which suggests that the resting excitability levels of the two hemispheres are different [12]. Not only are the motor pathways lateralized, but the integration from the sensory system also affects this lateralized model. Another TMS study found demonstrated that during action observation, the motor cortex is activated in contrast to the sensory information displayed [48]. During demonstration of a right-handed movement, the left motor cortex was more excitable following TMS stimulation, and during left-handed demonstration, the right motor cortex was more excitable [48,49]. This confirms that there are anatomical differences in the internal cortical representation between the two hemispheres, which correspond to laterality of function. It has also been suggested that, even though these specializations are present in the control of each limb, each hemisphere can draw on the preferred strategy of the opposite hemisphere to efficiently complete movements [1]. This is also reflected in the neurological data collected in this study. The N24 and N30 cortical peaks showed opposite directional and amplitude changes between hands, suggesting that the underlying structures that create these peaks are either different or working at different timelines in both hemispheres. 

The Non-Dom hand was significantly more accurate at baseline, and showed significant improvement, as did the Dom hand at both post- acquisition and retention. These results support studies where the Non-Dom limb was more accurate at performing a task [1,2,6,11]. The task used in the current study differs from the more gross motor control scheme required by reaching tasks used in previous works [1,2,6,11], as it required greater fine motor control for the tracing sinusoidal waves, due to the isolation of the thumb movement. The fact that these studies used a variety of different tasks, and the results were similar, suggests a globally adaptable control scheme (feedforward for the Dom limb, and feedback for the Non-Dom limb), that can be applied to both gross motor movements and fine motor movements. One study found that the Non-Dom limb was less accurate when completing a similar fine motor learning task, however, they used the first dorsal interosseous muscle (FDI) as opposed to APB [13]. Current technology design may favor the use of a muscle such as APB, which is more widely used for handheld electronics. This provide a more comparable outcome when measuring differences in performance between the limbs.

The greater baseline accuracy seen in the Non-Dom hand has at least two possible explanations. As previously stated, the Non-Dom limb is thought to use a feedback control strategy that centers around impedance control, which generally is an inefficient way to achieve a goal, however, the accuracy of this type of control is much higher than one that uses the predictive approach favored by the dominant limb [3]. Additionally, this work provided evidence to suggest the use of both types of control strategies in both limbs; however, it is the time it takes to switch from one to the other that causes differences in accuracy. Their work indicated that both the Dom and Non-Dom limb utilized predictive mechanisms at the onset of movement, however the Non-Dom limb switched to the feedback mechanism earlier than the Dom limb [3].

The other possible explanation for the increase in accuracy seen in the Non-Dom hand has to do with attention and complexity. Challenge point framework states that the level of functional difficulty of the task leads to difficulty learning [50], which would result in participants being closer to their optimal challenge point, thus leading to enhanced learning. Participants may have had a preconceived notion that performing the task with the Non-Dom hand would be more difficult than performing the task with the Dom hand, thus leading to an increase in concentration and attention to compensate. In most cases this increase in attention leads to a greater ability to learn and retain information required by the task [51]. This could explain the increase in accuracy during the initial stages of learning. Understanding the nature of these behavioral changes can perhaps be put into context in relation to the differential changes that were observed between groups within the aforementioned N24 and N30 peaks. On average, it can be seen that both groups demonstrated an increase in accuracy (decreased error) with time. Within the Non-Dom group, the increase in the N24 and decrease in the N30 following acquisition, which are shown to be associated with cerebellar input to S1 and motor planning, respectively, would suggest that there is an increase in cerebellar inhibition, perhaps due, in part, to the unfamiliarity of the Non-Dom hand being predominantly relied upon for skill learning, with regards to tracing, reaching or throwing. The juxtaposition of the peak changes observed for the Dom group following motor skill acquisition supports the initial reliance on feedforward control mechanisms of the right hand, given previous experience.

Within the current study, it is important to note that although there are similar behavioural changes paired with differential cortical peak changes observed for the Dom and Non-Dom hands, we are unable to speak to how these cortical peak changes may have changed following the 24 h period. Both groups demonstrate an improved accuracy compared to baseline during the retention task, however, SEPs were not measured during the retention session. A follow up study in which SEPs are measured during retention might aid in supporting the differences in feedforward and feedback mechanisms that are favored by the Dom and Non-Dom limbs respectively, and whether this trend is continued throughout the later stages of learning. Interestingly, previous work measuring motor evoked potential changes [40], in response to the same task employed here, found that all the MEP changes occurred post-acquisition, with no further changes at retention. This was thought to be due to the fact that changes in motor cortex reflect the early stage of learning (within the first few hours). The retention task in the current study was performed 24 h following the initial session, which may be considered soon by some, however studies have demonstrated that retention can occur in as little as 6–8 h following the initial session. Other studies have shown that retention may be optimal at up to 5–6 days following the initial session. The current time point of 24 h was chosen in an effort to ensure participant availability, however, a study in which the retention session is either performed about a week from the initial session, or that has two retention sessions (24 h and 1 week from the initial), would be beneficial for future work.

## 7. Conclusions 

This study found differential changes in SEP peak amplitudes and motor acquisition performance between the non-dominant and dominant limb. This is one of the few studies to compare novel learning between the limbs using neurological measures and the results suggest that there may be underlying neurological differences responsible for performance differences by stating the independent use of feedback and feedforward in the adaption to the left and right limbs, respectively. By developing techniques that compare the limbs and integrate the cortex into the equation, we can develop a new framework.
